# The association between cannabis use and outcome in pharmacological treatment for opioid use disorder

**DOI:** 10.1186/s12954-021-00468-6

**Published:** 2021-02-23

**Authors:** Tea Rosic, Raveena Kapoor, Balpreet Panesar, Leen Naji, Darren B. Chai, Nitika Sanger, David C. Marsh, Andrew Worster, Lehana Thabane, Zainab Samaan

**Affiliations:** 1 Department of Psychiatry and Behavioural Neurosciences, McMaster University, 100 West 5th St, Hamilton, ON L8N 3K7 Canada; 2 Department of Health Research Methods, Evidence, and Impact, McMaster University, Hamilton, ON Canada; 3 Bachelor of Health Sciences, McMaster University, Hamilton, ON Canada; 4 Neurosciences Graduate Program, McMaster University, Hamilton, ON Canada; 5 Department of Family Medicine, McMaster University, Hamilton, ON Canada; 6 Michael G. DeGroote School of Medicine, McMaster University, Hamilton, ON Canada; 7 Medical Sciences Graduate Program, McMaster University, Hamilton, ON Canada; 8 Northern Ontario School of Medicine, 935 Ramsey Lake Rd, Sudbury, ON P3E 2C6 Canada; 9 Canadian Addiction Treatment Centres, 175 Commerce Valley Drive West, Suite 300, Markham, ON L3T 7P6 Canada; 10 ICES North, 56 Walford Rd, Sudbury, ON P3E 2H2 Canada; 11 Health Sciences North Research Institute, 56 Walford Rd, Sudbury, ON P3E 2H2 Canada; 12 Department of Medicine, McMaster University, Hamilton, ON Canada; 13 Biostatistics Unit, Research Institute at St Joseph’s Healthcare, Hamilton, ON Canada; 14 Departments of Pediatrics/Anesthesia, McMaster University, Hamilton, ON Canada

**Keywords:** Marijuana, Cannabis, Opioid, Polysubstance use, Harm reduction

## Abstract

**Background:**

With the ongoing opioid crisis and policy changes regarding legalization of cannabis occurring around the world, it is necessary to consider cannabis use in the context of opioid use disorder (OUD) and its treatment. We aimed to examine (1) past-month cannabis use in patients with OUD, (2) self-reported cannabis-related side effects and craving, and (3) the association between specific characteristics of cannabis use and opioid use during treatment in cannabis users.

**Methods:**

Participants receiving pharmacological treatment for OUD (*n* = 2315) were recruited from community-based addiction treatment clinics in Ontario, Canada, and provided information on past-month cannabis use (self-report). Participants were followed for 3 months with routine urine drug screens in order to assess opioid use during treatment. We used logistic regression analysis to explore (1) the association between any cannabis use and opioid use during treatment, and (2) amongst cannabis-users, specific cannabis use characteristics associated with opioid use. Qualitative methods were used to examine responses to the question: “What effect does marijuana have on your treatment?”.

**Results:**

Past-month cannabis use was reported by 51% of participants (*n* = 1178). Any cannabis use compared to non-use was not associated with opioid use (OR = 1.03, 95% CI 0.87–1.23, *p* = 0.703). Amongst cannabis users, nearly 70% reported daily use, and half reported experiencing cannabis-related side effects, with the most common side effects being slower thought process (26.2%) and lack of motivation (17.3%). For cannabis users, daily cannabis use was associated with lower odds of opioid use, when compared  with occasional use (OR = 0.61, 95% CI 0.47–0.79, *p* < 0.001) as was older age of onset of cannabis use (OR = 0.97, 95% CI 0.94, 0.99, *p* = 0.032), and reporting cannabis-related side effects (OR = 0.67, 95% CI 0.51, 0.85, *p* = 0.001). Altogether, 75% of cannabis users perceived no impact of cannabis on their OUD treatment.

**Conclusion:**

Past-month cannabis use was not associated with more or less opioid use during treatment. For patients who use cannabis, we identified specific characteristics of cannabis use associated with differential outcomes. Further examination of characteristics and patterns of cannabis use is warranted and may inform more tailored assessments and treatment recommendations.

## Introduction

The health and policy landscapes of substance use and addiction are changing as jurisdictions around the world legalize recreational cannabis while facing an ongoing opioid crisis. With legalization, the prevalence of cannabis use is expected to rise [1, 2], raising particular concerns about its impact on individuals with existing psychiatric comorbidity. Understanding the impact of cannabis use for patients with opioid use disorder (OUD) in the light of high rates of concurrent use is important [3]. The continuing opioid crisis across North America is reflected in ongoing increases in opioid overdose deaths [4], as well as increased enrolment in medication-assisted treatment (MAT) [5, 6] , including treatment with methadone and buprenorphine–naloxone. MAT reduces opioid cravings and withdrawal to support abstinence from opioid use, and has been shown to improve outcomes including overall productivity and quality of life [7, 8]. However, outcomes in MAT are variable [9, 10], and ongoing examination of the impact of modifiable factors such as psychiatric comorbidity and polysubstance use on treatment is important.

The retention of patients in MAT can be hindered by the use of other psychoactive substances. Cocaine and alcohol use have been reported to negatively affect MAT outcomes [11, 12]; however, research surrounding the impact of cannabis on these outcomes is inconclusive. Whether or not cannabis use is associated with reduced opioid use has become a pressing clinical and scientific issue. Some studies report  that cannabis has a negative impact on MAT outcomes, such as increasing risk of relapse to opioid use [13]. However, other studies have reported no such impact [14, 15]. It is possible that several factors influence this relationship between cannabis and opioid use, such as geographical location, age, or sex [3, 16, 17]. For example, we have previously found that past-month cannabis use (i.e. cannabis use in the previous 30 days) was significantly associated with opioid use in women but not in men [3]. Furthermore, there has been conflicting evidence about the impact of cannabis use on opioid withdrawal symptoms. Some evidence has indicated that cannabis decreases opioid withdrawal symptoms such as pain, anxiety and sleep disturbances [18].

More information is required to better understand the impact of cannabis use on opioid use outcomes for patients with OUD and it may not merely be the presence of cannabis use or non-use that influences treatment outcomes, but rather, that particular characteristics of cannabis use play an important role. Specifically, the effect of cannabis dependence, craving, withdrawal, and side effects on outcomes in this population is understudied. It has been estimated that approximately 9% of individuals who try cannabis eventually become dependent users [19]. We have previously found that prevalence of cannabis use disorder amongst patients with OUD is 28% [20]. A better understanding of the impact of cannabis use in patients receiving treatment for OUD may allow for better tailored treatment plans and improved patient outcomes.

Our objective is to examine the association between cannabis use and opioid use within a cohort of 2315 patients receiving MAT for OUD. We aim to examine, generally, the association between any cannabis use (compared to non-use) and opioid use during treatment, and, more specifically, amongst cannabis users, the association between characteristics of cannabis use and opioid use. The objectives for our study are as follows, to:

1. Determine the prevalence of self-reported past-month cannabis use in patients treated for OUD;
2. Explore the association between any past-month cannabis use (versus no use) and opioid use (other than methadone or buprenorphine) during treatment;
3. Amongst cannabis users, assess cannabis use characteristics and the prevalence of cannabis-related side effects, cravings, frequency of use, and age of onset of cannabis use;
4. Amongst cannabis users, explore the association between cannabis use characteristics and opioid use during treatment;
5. Examine sex differences in the association between cannabis use characteristics and opioid use during treatment.

## Methods

### Data

Prospective observational data used in this study were collected in the Pharmacogenetics of Opioid Substitution Treatment Response (POST) study in a collaboration between researchers at McMaster University and the clinical programs of the Canadian Addiction Treatment Centres. Participants were recruited from 31 outpatient addiction clinics in Ontario, Canada, between May 2018 and January 2020, using the following inclusion criteria: patients diagnosed with OUD using *Diagnostic and Statistical Manual of Mental Disorders, Fifth Edition* (DSM-5) criteria and receiving outpatient methadone or buprenorphine–naloxone treatment [21]. Participants were enrolled in treatment for varying lengths of time prior to study recruitment (median 2.6 years).

Ethics approval was obtained, and the study was conducted in accordance with the ethical guidelines of the Hamilton Integrated Ethics Board (project ID 4556). Participants provided verbal and written informed consent and were able to withdraw from the study at any time. Our study procedures and analyses are reported in accordance with the Strengthening the Reporting of Observational Studies in Epidemiology (STROBE) guidelines [22].

### Study Measures

At the time of recruitment, participants completed comprehensive baseline assessments by trained interviewers, including demographic information, MAT treatment history, and self-reported cannabis use history. Past-month cannabis use (i.e. cannabis use in the previous 30 days) was assessed using the Maudsley Addiction Profile (MAP) [23]. Participants reporting past-month cannabis use were identified as “cannabis users”, and those denying past-month cannabis use were identified as “cannabis non-users”. Individuals identified as cannabis-users answered additional questions regarding their patterns of cannabis use. Cannabis craving was assessed using the 12-item Short Form Marijuana Craving Questionnaire (SF-MCQ), which assesses four components of cannabis cravings: compulsivity, emotional benefit, expectancy of positive outcomes through use, and purposefulness of cannabis [24]. Each component contains three corresponding items from original questionnaire that contained the greatest inter-item correlation and within-factor reliability assessed by Cronbach’s alpha coefficient [25]. Each item is scored on a 7-point Likert scale, from “strongly disagree” to “strongly agree”. The scores from the three items for each component are totalled to achieve a minimum score of 3 and a maximum of 21. Higher scores indicate increased craving [25]. Participants who reported having any past-month cannabis use were also asked to report whether they have experienced any side effects from their use, in response to the following question: “Have you had any side effects from using marijuana?”. Participants were able to select from the following prompted responses: “no side effects”, “sexual problems”, “weight gain”, “paranoia”, “hallucinations”, “lack of motivation”, “slower thought process”, “poorer school or work performance”, or “other”.

All participants who reported using cannabis also answered the open-ended question “What effect does marijuana have on your opioid substitution treatment?”. Qualitative analysis was conducted using Nvivo software (QSR International [Americas] Inc., Burlington, Massachusetts, USA) to identify common themes from patient answers [26]. We used Microsoft Excel (Microsoft Corporation, Redmond, Washington, USA) to review the free-text question data in order to minimize typographical errors in responses. We then imported data into Nvivo and subjected data to multiple queries and coding functions. We began by running the data through a word frequency query to identify patterns in the responses and improve analytic accuracy. Words with frequency weighting outputs greater than 0.5% were coded as nodes, and words with outputs ranging from 0.2 to 0.5% were scanned and included in established nodes. Word frequency queries were followed with text search queries to identify new words and stemmed variants that were then coded into established nodes or founded as new nodes. Nodes were refined as a greater number of words were coded and pattern and coding strategies were continuously developed. Refined nodes were labelled as themes. Response data led to the identification of 9 distinct “themes” or “effects” of cannabis on opioid substitution treatment: (1) reduction in cravings, (2) increased effects of MAT, (3) general negative effects, (4) uncertain of effects, (5) help to manage pain, (6) general positive effects, (7) help to taper dose, (8) reduction in withdrawal symptoms, (9) no impact.

Participants were followed in the study for 12 months with approximately weekly urine drug screens as per routine clinical protocol for morphine, oxycodone, fentanyl, cannabis metabolites, cocaine, amphetamine, methamphetamine, diazepam, methadone metabolite, and buprenorphine using the FaStep Assay (Trimedic Supply Network Ltd, Concord, Ontario, Canada) [27]. The use of urine drug screens for cannabis metabolites and amphetamines was variable between participating clinics. For the purposes of this study, we define “opioid use” as any non-methadone or buprenorphine opioid use detected by urine drug screens.

### Statistical analysis

**Fig. 1:**
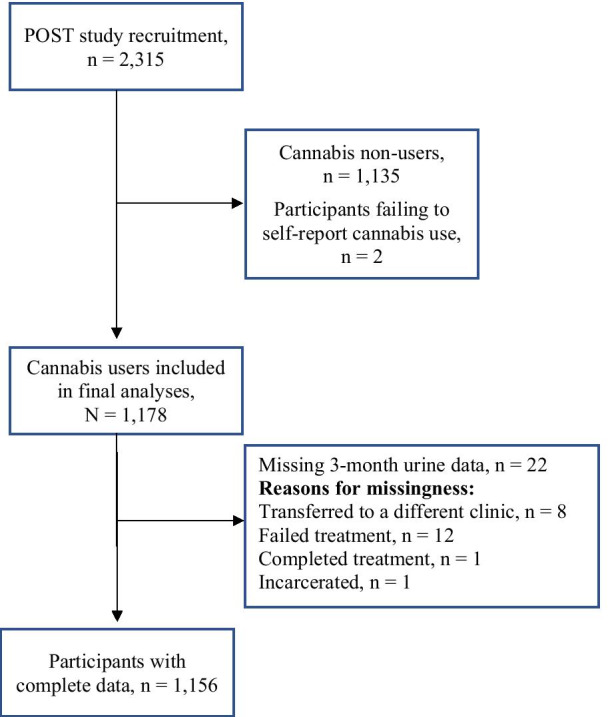
Study flow diagram

All quantitative analyses were conducted using Stata version 15.1 (StataCorp, College Station, Texas, USA). Our first objective was to determine the prevalence of self-reported past-month cannabis use in patients treated for OUD. Within our total study sample of 2315 participants, 51% reported past-month cannabis use and were identified as “cannabis users” (*n* = 1178). Please see Fig. 1, study flow diagram, for complete details.

Our second objective was to explore the association between any past-month cannabis use (versus no use) and opioid use during treatment. With this objective, we attempt to address the clinical question: “*Does cannabis use reduce opioid use?*”. We constructed a logistic regression model, with the dichotomous variable any opioid use (yes/no) in the 3 months following study entry as the dependent variable. The 3-month time frame was chosen as it aligned most closely with the responses provide about past-month cannabis use at study entry (i.e. cannabis use was reported for one month prior to study entry, and opioid use was then assessed for three months immediately following). The variable of interest tested in this model was past-month cannabis use status (yes/no), and the model was adjusted for factors with known association with opioid use including age, sex, type of MAT (methadone versus buprenorphine–naloxone), medication dose, and length of time in treatment. 

Our third objective was to assess cannabis use characteristics and the prevalence of cannabis-related side effects, cravings, frequency of use, and age of onset of use amongst cannabis users. We summarized baseline characteristics at the time of study entry for the cannabis user population using mean values with standard deviation (SD) or median values with first and third quartiles for continuous variables, as appropriate. We summarized dichotomous variables using frequencies and percentage.

Our fourth objective was to explore the association between different cannabis use characteristics, cannabis-related side effects, cravings, frequency of use, and opioid use during treatment. We constructed a logistic regression model with the dichotomous variable any opioid use (yes/no) in the 3 months following study entry as the dependent variable. The covariates of interest tested in this model included daily versus less than daily cannabis use, age of onset of cannabis use, side effects from cannabis (yes/no), and the Marijuana Cravings Questionnaire total score. This model was also adjusted for the above described factors including age, sex, type of MAT, dose, and length of time in treatment. Our sample size of 1178 cannabis users, with an event rate of 45% and 527 participants with opioid use at 3 months, is adequate [28]. Results of our logistic regression analysis are reported using odds ratios (OR) with 95% confidence intervals (CI). We present the estimates of effect for our main variables of interest in the results table and present all variables adjusted for in a footnote in the table. Our final objective was to examine between sex and within sex differences in the association between cannabis use characteristics and opioid use during treatment. To examine whether sex moderates the association between cannabis use characteristics and opioid use, we conducted a moderation analysis by sex, adding interaction terms between our cannabis use characteristics of interest and sex. To examine whether there are different associations between cannabis use characteristics of interest based on sex, we conducted subgroup analysis by sex. Sex was operationalized according to biological sex at birth for the purposes of this study.

Missing 3-month urine data affected 22 participants (2%) and reasons for missingness included transfer to another clinic (*n* = 8), failed treatment (*n* = 12), completed treatment (*n* = 1), and incarceration (*n* = 1; see Fig. 1). Due to the low percentage of missing data, missingness was handled by available case analysis. We also planned one a priori subgroup analysis by sex, based on our previous finding of sex differences in cannabis use and its impact in MAT in a previous study [3].

## Results

Past-month cannabis use was reported by 51% of our total study sample (*n* = 1178). In Table 1, we present demographic and clinical and cannabis-use characteristics for participants reporting past-month cannabis use and for those denying past-month cannabis use. Amongst self-reported cannabis users, mean age was 37.7 years (SD = 10.6) and 40% of these participants were female. Methadone was more commonly prescribed (80%). Altogether, 46% of cannabis-users had opioid use at 3 months as evidenced by positive urine drug screens. We did not identify a significant association between self-reported cannabis use and opioid use for 3 months following study entry, adjusting for patient age, sex, type of MAT, dose, and length of time in treatment (OR = 1.03, 95% CI 0.87–1.23, *p* = 0.703).

**Table 1:** Demographic, clinical, and cannabis-use characteristics of patients with past-month cannabis use

Characteristic	Total sample (*N* = 2313)	Cannabis non-user (*n* = 1135)	Cannabis user (*n* = 1178)
Demographic			
Age in years; mean (SD)	39.3 (10.9)	40.9 (10.9)	37.7 (10.6)
Female sex; *n* (%)	1025 (44.3%)	557 (49.1%)	468 (39.7%)
Married; *n* (%)	673 (29.1%)	356 (31.4%)	317 (26.9%)
Unemployed; *n* (%)	1548 (66.9%)	761 (67.1%)	787 (66.8%)
Clinical			
Length of time in treatment, in years; median (Q1, Q3)	2.6 years (0.83, 6)	3 years (0.92, 7)	2 years (0.75, 6)
Type of treatment; *n* (%)			
Methadone	1833 (79.4%)	890 (78.6%)	944 (80.1%)
Buprenorphine–naloxone	477 (20.7%)	243 (21.5%)	234 (19.9%)
Medication dose in mg/day; mean (SD)			
Methadone	70.4 mg (40.6)	72.9 mg (41.3)	68.1 mg (39.8)
Buprenorphine–naloxnaloxone	12 mg (6.8)	12 mg (7.0)	12 mg (6.6)
Opioid use at 3 months^a^; *n* (%)	1015 (44.8%)	488 (44.0%)	527 (44.7%)
Percentage of opioid-positive urine drug screens amongst users; median (Q1, Q3)	25 (11.1, 50)	30 (12.5, 55.6)	21.4 (10, 50)

*SD* standard deviation, *MAT* medication-assisted treatment, *Q1* 25th percentile, *Q3* 75th percentile

^a^Data available for *n* = 1155 participants

For patients reporting past-month cannabis use, daily use was common (68%) and approximately half of the participants reported experiencing side effects from their cannabis use (Table 2). Many participants reported that cannabis use has no effect on their MAT (75%); meanwhile, fewer participants reported that cannabis use helps with opioid cravings (6.9%) and opioid withdrawal (8.3%). Few participants reported feeling that cannabis use had a negative impact on MAT and worsened OUD symptoms (2.4%).

**Table 2:** Cannabis use characteristics amongst patients reporting past-month cannabis use (*n* = 1178)

Cannabis use characteristic	Statistic
Daily use; *n* (%)	798 (67.7%)
Age of first cannabis use in years; mean (SD)	14.2 (4.5)
Age at first regular cannabis use in years (defined as use at least twice monthly); mean (SD)	16.7 (6.9)
Self-reported cannabis side effects; *n* (%)	583 (49.5%)
Type of side effects reported amongst individuals reporting cannabis-related side effects^a^; *n* (%)	
Sexual problems	2 (0.3%)
Weight gain	28 (4.8%)
Paranoia	62 (10.6%)
Hallucinations	10 (1.7%)
Lack of motivation	101 (17.3%)
Slower thought process	153 (26.2%)
Decreased school/work performance	86 (14.8%)
Other	141 (24.2%)
Marijuana cravings score; mean (SD)	37.4 (16.3)
Self-reported impact of cannabis on MAT	
No impact	881 (74.9%)
Helps with opioid cravings	81 (6.9%)
Helps with opioid withdrawal symptoms	98 (8.3%)
Helps with MAT dose	14 (1.2%)
Helps with pain management	17 (1.4%)
Increases effect of MAT	31 (2.6%)
Other positive effects	82 (7%)
Worsens OUD symptoms	28 (2.4%)
Unsure	15 (1.3%)

*SD* standard deviation, *MAT* medication-assisted treatment, *OUD* opioid use disorder

^a^Participants were able to report only one primary side effect experienced

The results of our logistic regression analysis examining the association between cannabis use characteristics and opioid use are presented in Table 3. We found that amongst cannabis users, those who use cannabis daily are less likely to have opioid use than people who use cannabis occasionally (OR = 0.60, 95% CI 0.46–0.78, *p* < 0.001). This association was present for both men and women. Older age of onset of cannabis use was associated with lower odds of opioid use (OR = 0.97, 95% CI 0.94, 0.99, *p* = 0.032). Patients using cannabis who report having cannabis-related side effects were less likely to have opioid use compared to patients who do not report any side effects (OR = 0.66, 95% CI 0.52, 0.84, *p* = 0.001).

**Table 3:** Multivariable model of cannabis-use characteristics associated with opioid use, amongst cannabis users (*n* = 1154)

Characteristic	OR	95% CI	*p*
Age of onset of cannabis use in years (for every 1 year increase in age)	0.97	0.94, 0.99	0.032
Non-daily cannabis use	[reference]		
Daily cannabis use	0.60	0.46, 0.78	< 0.001
Marijuana Cravings Questionnaire Score (for each 10-point increase)	1.06	0.98, 1.14	0.144
No side effects from cannabis reported	[reference]		
Side effects from cannabis reported	0.66	0.52, 0.84	0.001

*OR* odds ratio, *CI* confidence interval

Model is adjusted for age, sex, type of MAT, dose, and years in treatment

Interaction analysis revealed no significant moderating effect of sex on our cannabis use characteristics of interest (age of onset of cannabis use by sex: OR = 0.99, 95% CI 0.94, 1.05, *p* = 0.725; daily cannabis use by sex: OR = 0.92, 95% CI 0.53, 1.57, *p* 0.748; side effects from cannabis by sex: OR = 1.53, 95% CI 0.93, 2.50, *p* = 0.092; marijuana cravings score by sex: OR = 1.01, 95% CI 0.99, 1.03, *p* = 0.100). Using subgroup analysis by sex, we found the association between reporting cannabis-related side effects and lower odds of opioid use to hold for men (OR = 0.55, 95% CI 0.40, 0.75, *p* < 0.001), but not for women (OR = 0.86, 95% CI 0.59, 1.26, *p* = 0.442). Additionally, for women, but not men, higher marijuana cravings score was associated with increased odds of opioid use (scaled for each 10-point increase in score: OR = 1.14, 95% CI 1.01, 1.28, *p* = 0.034).

## Discussion

Whether cannabis use reduces opioid use in patients with OUD is an important clinical and scientific question. In this study of 2315 patients treated for OUD, we did not detect a significant positive or negative association between any past-month cannabis use compared to no past-month cannabis use and opioid use. Owing to the little consensus in the literature about the impact of cannabis use in OUD treatment [16], we sought to explore in greater detail specific characteristics of cannabis use associated with treatment outcomes. Cannabis use is common amongst individuals with OUD (as evidenced by a 51% prevalence of cannabis use identified in our study); however, not all cannabis use may be considered equal. Therefore, we were interested in exploring characteristics of cannabis use that may be associated with treatment outcomes in this population: higher frequency (i.e. daily use), age of onset of cannabis use, experience of side effects, and experience of craving.

We found that, amongst patients using cannabis, daily cannabis use was associated with lower odds of opioid use during treatment than occasional cannabis use.

Frequency of cannabis use has previously been investigated as an important factor in the experience of patients with OUD. Another study found that cannabis users scored lower on the Clinical Opiate Withdrawal Scale [29] in comparison with non-cannabis users, and the frequency of cannabis use was inversely proportional to the severity of withdrawal symptoms, which coincides with the relationship found in this study [30]. These findings may also be considered in light of patients’ self-reported experience of cannabis use reducing OUD-related symptoms such as craving and withdrawal; however, only about 15% of participants self-reported that cannabis use helped with their opioid craving or withdrawal. While daily cannabis use may provide some benefit for MAT outcomes, additional research has shown that daily cannabis users are more likely to report anxiety symptoms in comparison with occasional cannabis users [31, 32]. Better understanding the risks and benefits of frequent cannabis use in this population is necessary. Its impact on mental health symptoms, pain, and quality of life in this population is unclear. Nearly 50% of participants in our study reported experiencing negative side effects from cannabis, including impact on cognition, motivation, as well as work and school performance. Examining the association between cannabis use and social functioning outcomes in OUD treatment is equally important.

Older age of first cannabis use was associated with lower odds of opioid use during treatment. Younger age of onset of substance use has been associated with polysubstance use, higher severity of substance use disorder, and worse outcomes in treatment [33, 34]. Additionally, the deleterious effects of cannabis use on neurodevelopment with younger age of onset may lead to worse outcomes in adulthood [35].

We also found an association between self-reported cannabis side effects and lower odds of opioid use. Whether this finding represents a protective effect of cannabis side effects or a reporting bias is unclear. Patients who have insight into their substance use, including side effects, and are forthcoming with this information may have better outcomes in treatment overall [36]. On the other hand, patients commonly self-report fewer side effects experienced with cannabis use in comparison with medication used in MAT [37]. Patients may choose to substitute cannabis for various prescription drugs, such as anti-depressant or anti-anxiety medications [38], or illicit drugs including cocaine [38], due to perceived lower side effects [39]. Finally, it is also possible that individuals who are using opioids may be less aware of cannabis’ side effects or may attribute these to the opioids used rather than to cannabis.

Subgroup analysis by sex revealed sex differences in the association between cannabis use characteristics and opioid use outcome. We found that for men with past-month cannabis use, reporting side effects from cannabis use was associated with lower odds of opioid use. For women, reporting higher cannabis cravings was associated with higher odds of opioid use. It is important to consider possible sex differences in the patients’ experience of side effects. In a study by Cuttler et al., during cannabis intoxication, men were reported to experience increased appetite, while women were more likely to report loss of appetite [39]. During withdrawal, men were more likely to report insomnia and vivid dreams, while women reported more nausea and anxiety [39]. Women appear to experience, or report, more negative side effects from substance use, which is a common trend found in cannabis, cocaine, and heroin use [40]. Additionally, women may be more likely to experience adverse effects from medications used to treat substance use disorders [40].

Finally, considering patients’ perspectives on the impact of their cannabis use is also important. Patient-centered care focuses on the patient’s experience by exploring their ideas and feelings about their illness [41]. This approach to care allows for physicians and patients to find common ground, where physicians may better understand and respond to the needs of their patients [42]. We aimed to use a patient-centered approach by directly asking participants what impact they believe cannabis has on their treatment. Their responses along with additional anecdotes that participants share about their experiences can be incorporated into research in this field, to gain a holistic understanding of the overall impact of cannabis on OAT outcomes. These responses, when incorporated into patient care, may also lead to increased patient satisfaction [43].

Future studies should further examine specific characteristics and patterns of cannabis use that may be protective or problematic in MAT. The study of the impact of cannabis is further complicated by the fact that cannabis and its derivatives are available for consumption in different combinations, concentrations and mixed or contaminated with other psychoactive agents. As research on cannabis evolves, one such characteristic to consider may be primary cannabis strain used. The use of cannabis strain may differ depending on the purpose of use. It was found that hybrid strains were the most commonly preferred strain amongst individuals using cannabis for pain treatment, and Indica strains have been preferred for insomnia or sleep disorders [44]. Strains vary in the quantity of cannabinoid molecule cannabidiol (CBD) found, which may impact the overall effects of cannabis. Research has shown that CBD assists with reducing rewarding components of drug abuse, such as in cases of cocaine or amphetamine abuse [45]. CBD itself may also have a lower risk for abuse, as researchers found limited rewarding or reinforcing effects of CBD in rodents [45]. These findings create plausibility for strains with high CBD content to serve as a protective factor in reducing risk of opioid use in patients with OUD.

Our study is strengthened by its observational design, including a large cohort of patients who are representative of the general MAT population and were recruited using a multi-site design. We present detailed information on cannabis use characteristics, broadening the general understanding of cannabis use patterns in this population, and we include a qualitative analysis that gives regard to the important, but too often overlooked, patient perspective. Limitations include the use of self-report cannabis data, increasing the risk of reporting biases; however, we have previously found that the sensitivity and specificity of cannabis self-report is 79.9% (95% CI 72.7–85.8) and 80.0% (95% CI 73.6–85.4) in the OUD population, assessed using concordance with urine drug screen results [3]. Importantly, generalizability and relevance of this study may be limited in settings in which MAT takes on a firmly abstinence-based role, such that retention in treatment is contingent on abstinence from substance use. However, in the setting of a harm-reduction treatment, understanding the relationship between opioid and non-opioid substance use in treatment is particularly important. Finally, assessment of the relationship between cannabis and opioid use in this population over a longer time period will be an important area for future research.

## Conclusion

We did not find an association between cannabis use and opioid use in OUD treatment. However, we demonstrate that cannabis use is not benign; numerous patients in this study reported cannabis-related side effects and cravings. For patients using cannabis during treatment, we provide evidence that certain characteristics of cannabis use are associated with less opioid use, including daily use, and reporting cannabis-related side effects (for men). Other characteristics of cannabis use are associated worse outcomes, including younger age of onset of cannabis use and cannabis cravings (for women). Continuing to examine of the impact of cannabis use in OUD treatment remains important. Furthering our understanding of patterns and characteristics of cannabis use that may be more or less harmful may allow healthcare providers to tailor assessment and treatment accordingly in order to support better patient outcomes.

## Data Availability

The datasets used and/or analysed during the current study are available from the corresponding author on reasonable request.

## Abbreviations

OUD,: Opioid use disorder
MAT,: Medication-assisted treatment
CBD,: Cannabidiol
MAP,: Maudsley Addiction Profile
SF-MCQ,: Short Form Marijuana Craving Questionnaire
SD,: Standard deviation
Q1,: 25^Th^ percentile
Q3,: 75^Th^ percentile
OR,: Odds ratio
CI,: Confidence interval
